# Genetic diversity and distribution of noroviruses among all age groups of patients with diarrhea in Amhara National Regional State, Ethiopia

**DOI:** 10.1371/journal.pone.0303887

**Published:** 2024-05-21

**Authors:** Dessie Tegegne, Aschalew Gelaw, Dawit Hailu Alemayehu, Tamrayehu Seyoum, Dereje Leta, Getachew Ferede, Andargachew Mulu, Baye Gelaw

**Affiliations:** 1 Department of Medical Microbiology, School of Biomedical and Laboratory Sciences, College of Medicine and Health Sciences, University of Gondar, Gondar, Ethiopia; 2 Department of Medical Laboratory Sciences, College of Health Sciences, Debre Tabor University, Debre Tabor, Ethiopia; 3 Armauer Hansen Research Institute, Addis Ababa, Ethiopia; 4 Ethiopian Public Health Institute, Addis Ababa, Ethiopia; College of Health Sciences, School of Medicine, Abibas Ababa University, ETHIOPIA

## Abstract

**Background:**

Norovirus (NoV) is the leading cause of diarrheal disease worldwide and the impact is high in developing countries, including Ethiopia. Moreover, there is a significant and fluctuating global genetic diversity that varies across diverse environments over time. Nevertheless, there is a scarcity of data on the genetic diversity of NoV in Ethiopia.

**Objective:**

This study was aimed to assess the genetic diversity and distribution of NoVs circulating in the Amhara National Regional State, Ethiopia, by considering all age groups.

**Methods:**

A total of 519 fecal samples were collected from diarrheal patients from May 01/2021 to November 30/ 2021. The fecal samples were screened for the presence of NoVs using real-time RT-PCR by targeting a portion of the major capsid protein coding region. The positive samples were further amplified using conventional RT-PCR, and sequenced.

**Results:**

The positivity rate of NoV was (8.9%; 46/519). The detection rate of NoV genogroup II (GII) and genogroup I (GI) was 38 (82.6%) and 8 (17.4%), respectively. Overall, five distinct GII (GII.3, GII.6, GII.10, GII.17, and GII.21) and two GI (GI.3 and GI.5) genotypes were detected. Within the GII types, GII.3 was the predominant (34.2%) followed by GII.21 (15.8%), GII.17 (10.5%), GII.6 and GII.10 each (2.6%). Norovirus GII.21 is reported for the first time in Ethiopia. The genetic diversity and distribution of NoVs were significantly different across the four sampling sits and age groups. The phylogenetic analysis revealed close relatedness of the current strains with published strains from Ethiopia and elsewhere.

**Conclusion:**

The distribution and genetic diversity of NoV was considerably high, with predominance of non-GII.4 genotypes. The GII.21 genotype is a new add on the growing evidences on the genetic diversity of NoVs in Ethiopia. Future nationwide surveillance studies are necessary to gain comprehensive data in Ethiopia.

## 1. Introduction

Diarrheal disease has been reported as a major public health problem globally [1]. They are the common causes of morbidity next to lower respiratory diseases in Sub-Saharan countries, including Ethiopia [2]. Viruses are the major cause of diarrheal diseases both in developed and developing countries [3]. Noroviruses (NoVs) are recognized as the leading cause of outbreaks and sporadic gastroenteritis among all ages worldwide, accounting for about 18% of the cases [4].

NoVs are genetically diverse groups of non-enveloped, single-stranded positive-sense RNA viruses belonging to the family *Caliciviridae* [5]. The genome is approximately 7.5 kb long and organized in three open reading frames (ORF). The ORF-1 encodes six non-structural proteins including the RNA-dependent RNA polymerase (RdRp), whereas ORF-2 and ORF-3 encode the major capsid protein (VP1) and minor capsid protein, respectively [6]. Genetic recombination and mutation are responsible for the existing diversity of NoVs [7]. Based on the variations on the VP1 region, NoVs are classified into 10 genogroups (GI-GX) and 49 genotypes, with genogroups I and II being frequent causes of infection in humans and NoV GIV rarely [8]. Of these, GII is by far the most predominant genogroup, responsible for about 70–90% of the cases [9,10]. Similarly, the GII.4 genotype has been reported as the predominant genotype [11,12]. However, in recent years, an increasing prevalence of non-GII.4 genotypes have been reported [13,14].

The genetic diversity of NoVs has become dynamic [13,15]. Because of this, there is no effective vaccine against NoV infections [16]. Hence, updated molecular data on the genetic diversity and distribution of NoV genotypes is important. The few available studies on NoV infection in sub-Saharan Africa were mainly focused on under-5 children [9,12,17]. Therefore, this study was designed to assess the genetic diversity and distribution of NoVs among patients of all ages with diarrhea attending at health institutions found in Gondar, Bahir Dar, Debre Markos and Debre Tabor areas, Amhara National Regional State, Ethiopia.

## 2. Materials and methods

### 2.1. Study settings and participants

The study was conducted at the health institutions of the four study sites, namely, Bahir Dar, Gondar, Debre Tabor, and Debre Markos, which are located in the Amhara National Regional State, Ethiopia. Each study site included two health centers and one comprehensive specialized hospital. A cross-sectional study was conducted from May 01/2021 to November 30/2021. The study period encompasses months of the rainy season in Ethiopia where the episodes of diarrheal diseases usually reach at its peak [18]. Those patients with diarrhea available in each of the selected health facilities during the study period were considered the study participants.

### 2.2. Sample size and sampling technique

The sample size was calculated using a single population proportion formula (n = Z α/22*P (1-P)/d2), where Zα/2 taken as 1.96 at 95% confidence interval (CI); P is the proportion taken from the previous study (13.3%) [12]; d is the desired level of precision (3%). By adding a 10% non-response rate, the total sample size was 550 diarrheic patients. This sample size was proportionally distributed for each study site based on the flow of average diarrheal disease cases during the previous years. Based on this analysis, 178, 152, 120, and 100 participants were distributed for each of the Bahir Dar, Gondar, Debre Markos, and Debre Tabor sampling sites, respectively. The study participants from each health facility were selected using a systematic random sampling technique. The sampling frame was taken from the previous year’s average monthly record of patients with diarrhea (1727). The sampling fraction (K) was analyzed by dividing the sampling frame to the allocated sample size. That is, 1727/550 = 3.14. Based on this calculation, the value of K was 3, and a sample was collected from every 3 patients with diarrhea in each health facility. The first study participant was selected by the lottery method among the list from one to three. The next study participant was identified systematically at every third interval until the required sample size was achieved.

### 2.3. Fecal sample collection technique

Five mL of fecal samples were collected from self-reporting diarrheal patients. Following collection, the samples were stored at -20 °C at the study sites. The samples were then transported on ice with a cold chain system to the Amhara Public Health Institute (APHI) for primary detection of NoV and longer-term storage of the samples at -70 °C that was used for further sequence analysis.

### 2.4. RNA extraction and norovirus detection

Ten percent stool suspensions were prepared with 1% phosphate buffered saline (PBS). Briefly, 100 μL of fecal samples were added to a labeled tube, and 900 μL of PBS was added and vortexed for 1 minute to make the suspension, and then centrifuged at 12,000 × g for 7 min. The supernatant was transferred to a clean 1.5 ml microcentrifuge tube and centrifuged for another 7 minutes at 12,000 × g. About 300 uL of the supernatant was transferred to a new labeled tube that can be used for ribonucleic acid (RNA) extraction. Ribonucleic acids were extracted using an automated RNA extraction method (BIOR automated system, German) following the manufacturer’s instructions. This automated system uses extraction kits that work on the principle of binding the nucleic acids with the paramagnetic beads and the removal of proteins and other contaminants by several washing steps. The nucleic acids are eluted off the beads using an elution buffer containing low concentrations of Tris-HCl buffer and EDTA, whereas paramagnetic beads remain captured on magnets in the kit. About 50 uL of the eluted RNA was used for amplification.

Norovirus screening was performed using a real-time reverse transcription polymerase chain reaction (rRT-PCR) with a 7500 real-time PCR system (Applied Biosystems, Foster City, CA, USA). Two sets of genogroup-specific primers, NV192 (forward) and NV193 (reverse) for GI, COG2F and COG2R for GII, and genogroup-specific probes (data in the S1 Table), were used as described previously [12,19]. Amplification and detection were done using the Quant Studio^™^ 5 rRT-PCR instrument (Applied Biosystems, USA). The final volume for each reaction was 25 μl. Each reaction contained 15 μl of one-step RT-PCR, 2X enzyme mix (reverse transcriptase, Taq polymerase, dNTPs, MgCl_2_, buffer, and enhancer), 1 μl of 10 μM each of the forward and reverse primers, 0.5 μl of a 10 μM probe, and 4.5 μl nuclease-free water. Finally, a 3 μl RNA template was added. The amplification condition was adjusted as follows: reverse transcription for 10 min at 45°C and initial denaturation for 10 min at 95°C, followed by 40 cycles of amplification (for 15 seconds at 95°C), and final elongation for 1 minute at 60°C as done previously [20]. The reaction was interpreted as positive when the threshold cycle (C_t_) values were less than 40.

### 2.5. Genotyping of noroviruses

All the positive fecal samples were transported using a cold chain system to the Armauer Hansen Research Institute (AHRI), Addis Ababa, for further amplification, purification, and library preparation. In order to further extract viral RNA, a 10% suspension of the fecal sample was also prepared in 1% PBS. Nucleic acid extraction was carried out using an automatic RNA extraction machine, as was done for the detection of NoV. Conventional RT-PCR was done using Superscript IV reverse transcriptase (Life Technology, California, USA). Previously published sets of primers, G1SKF/G1SKR and G2SKF/G2SKR, were used to amplify about 330 and 344 base-long fragments of the VP1 regions of GI and GII NoVs, respectively (data S1 Table), and done as described previously [21]. The amplification and reaction conditions of the RT-PCR were conducted as previously done [22]. Briefly, the reaction was adjusted as follows: RT at 50°C for 30 minutes and denaturation at 95°C for 2 minutes, followed by 35 cycles of 30 seconds at 94°C, 30 seconds at 48°C, and 1 minute at 72°C. Finally, an elongation step was performed for 10 minutes at 72°C. The PCR products were subjected to gel electrophoresis using 1.5% agarose and interpreted using a gel doc detection system (Bio-Rad, USA).

Gel bands corresponding to the desired NoV GI and GII amplicons were excised and then purified using gel purification method (Invitrogen, Sweden). Once the PCR products were purified, sequencing reaction was performed at the Ethiopian Public Health Institute (EPHI) using BigDye^®^ Terminator v3.1 Cycle Sequencing Kit) and sequencing was performed by using ABI Prism 3500 Genetic Analyser^®^ (Applied Biosystems, Foster City, CA, USA).

### 2.6. Phylogenetic analysis

Phylogenetic analysis was done using Molecular Evolution Genetic Analysis (MEGA7) [23]. Multiple sequence alignments were performed using the ClustalW sequence alignment algorithm. Phylogenetic trees were computed and constructed using the maximum likelihood method [24]. The percentage of replicate trees in which the associated taxa that were clustered together in the bootstrap test (1000 replicates) has been shown next to the branches. Norovirus GI and GII sequences were submitted and deposited in the GenBank database under the accession numbers OR367325-OR367328 (data in the S1 File) and OR793019-OR793043 (data in the S2 File), respectively.

### 2.7. Statistical analysis

Data were entered and analyzed with SPSS version 23 software (IBM, Chicago). A descriptive analysis was performed and presented using figures and tables. A chi-square test was conducted to see the association between dependent and independent variables. Variables with a P-value < 0.05 were considered statistically significant.

### 2.8. Ethics approval and informed consent

The study was conducted according to the guidelines of the Declaration of Helsinki and was approved by the University of Gondar institutional review board with a reference number of V/P/RCS/05//765/2021.

Written informed consent and assent were obtained from study participants and their parents, respectively. The participants had the right not to participate or withdraw from the study at any time (data in the S1 Appendix).

## 3. Results

### 3.1. Characteristics of participants and prevalence of noroviruses

Out of the 550 diarrheal patients approached, 519 agreed to participate in the study and provided samples, with a response rate of 94.4%. The ages of study participants ranged from 3 months to 85 years, with a mean age of 20 years. The number of female participants was 266 (51.3%). The proportion of study participants from Bahir Dar, Gondar, Debre Markos, and Debre Tabor were 178 (34.3%), 142 (27.3%), 100 (19.3%), and 99 (19.1%), respectively.

Norovirus RNA was detected in 46 fecal samples collected from the 519 diarrheal patients (8.9%; 95% CI: 6.6–11.6). The detection rate of NoV showed a statistically significant difference across the different age groups, with the highest prevalence observed among the elderly (33.3%; P<0.001), followed by children below the age of 5 years (12.5%; P<0.001). The proportions of NoV positive cases were also variable across the study settings. The detection rate of NoV was higher in Debre Tabor (17.2%) as compared to Bahir Dar (8.4%), Gondar (7%), and Debre Markos (4%) (P = 0.04). However, the NoV positivity rate was not significantly different between females and males (P = 0.4) (Table 1).

**Table 1:** Demographic characteristics and NoV positivity rates of diarrheic patients in Amhara National Regional State, May 2021 to November 2021.

Variables	Frequency (%)	Norovirus status		χ2 (P-value)
		Positive (%)	Negative (%)	
**Gender**				
** Male**	253 (48.7)	25 (9.9)	228 (90.1)	0.6 (0.400)
** Female**	266 (51.3)	21 (7.9)	245 (92.1)	
**Age in years**				
** <5**	160 (30.8)	20 (12.5)	140 (87.5)	31.4 (0.00)
** 4–17**	110 (21.2)	9 (8.2)	109 (91.8)	
** 18–64**	225 (43.4)	9 (4.0)	216 (96.0)	
** >64**	24 (4.6)	8 (33.3)	16 (66.7)	
**Study Settings**				
** Debre Tabor**	99 (19.1)	17 (17.2)	82 (82.8)	12 (0.040)
** Bahir Dar**	178 (34.3)	15 (8.4)	163 (91.6)	
** Gondar**	142 (27.3)	10 (7.0)	132 (93.0)	
** Debre Markos**	100 (19.3)	4 (4.0)	96 (96.0)	
**Residence**				
** Urban**	392 (75.5)	33 (8.4)	359 (91.6)	0.4 (0.60)
** Rural**	127 (24.5)	13 (10.2)	114 (89.8)	

### 3.2. Genotypic profiles of noroviruses

The average Ct-values for NoV GII and GI-positive samples were 29.2 and 32.5, respectively. Most of the NoV-positive samples with a C_t_-value less than the average were successfully genotyped. On the other hand, most of the NoV-positive samples with Ct-values greater than the average were not genotyped. Based on the partial VP1 gene sequence analysis, 29 out of the 46 positive samples (63.0%) were successfully genotyped. Of these genotyped samples, the genotyping rates of NoV GII and GI were 25 (65.8%) and 4 (50%), respectively. The sequenced NoVs belong to seven distinct genotypes, with the highest genetic diversity observed in GII. Norovirus GII.3 was the predominant genotype, followed by GII.21 and GII.17. Moreover, GII.21 is detected for the first time in Ethiopia. Noroviruses GI.3 and GI.5 were also detected, with the latter being relatively predominant (Table 2).

**Table 2:** Norovirus genogroups and genotypes detected from diarrheic patients in Amhara National Regional State, May 2021 to November 2021.

Noroviruses	Frequency	Percentage	Average C_t_-value
**Genogroup I**	8	17.4	**32.5**
**GI.3**	1	12.5	30
**GI.5**	3	37.5	29.5
**Untypable**	4	50.0	35.5
**Genogroup II**	38	82.6	**29.2**
**GII.3**	13	34.2	27.6
**GII.6**	1	2.6	29.5
**GII.10**	1	2.6	22.6
**GII.17**	4	10.5	26.15
**GII.21**	6	15.8	25.95
**Untypable**	13	34.2	33.78
**Total**	46	100	**30.0**

### 3.3. Geographic and age distribution of norovirus genotypes

Five of the seven distinct NoV genotypes (GII.3, GII.6, GII.17, GII.21, and GI.3) were detected in fecal samples collected in Bahir Dar. The dominant genotypes, GII.3 and GII.21, were detected from fecal samples obtained from participants from both Debre Tabor and Bahir Dar. Sporadic type of GII.6 and GII.10 genotypes were detected in samples from Bahir Dar and Gondar, respectively. Similarly, sporadic type GI.3 genotype was detected in a fecal sample collected in Debre Markos, whereas GI.5 genotypes were detected in samples obtained from Gondar and Bahir Dar. Whereas nearly half of the GII.3 and majority of the GII.21 genotypes were identified in fecal samples taken from under-five children, three-fourths of the GII.17 genotypes were detected among the elderly study participants. The diversity and distribution of NoV genotypes showed statistically significant differences across the study settings (P = 0.01) and age categories (P = 0.03) (Table 3).

**Table 3:** Distribution of NoV genotypes across demographic characteristics and study settings of participants in Amhara National Regional State, May 2021 to November 2021.

Variables	Norovirus genotypes (%)									Total	χ2 (P-value)
	GII.3	GII.21	GII.17	GII.6	GII.10	GII*	GI.3	GI.5	GI*		
**Settings**											
**Debre Tabor**	6 (46.2)	4 (66.6)	0	0	0	6 (46.0)	0	0	1 (25.0)	17 (17.2)	42.5 (0.01)
**Bahir Dar**	6 (46.2)	1 (16.7)	3 (75.0)	1 (100)	0	3 (23.0)	0	1 (33.3)	0	15 (8.4)	
**Gondar**	0	1 (16.7)	0	0	1 (50.0)	4 (31.0)	0	2 (66.7)	2 (50.0)	10 (7.0)	
**Debre Markos**	1 (7.6)	0	1 (25.0)	0	0	0	1 (100)	0	1 (25.0)	4 (4.0)	
**Age in years**											
**<5**	6 (46)	4 (66.6)	1 (25.0)	0	0	7 (54.0)	1 (100)	2 (66.7)	1 (25.0)	22 (13.8)	38.9 (0.03)
**5–17**	1 (8)	1 (16.7)	0	0	0	6 (46.0)	0	0	1 (25.0)	9 (8.2)	
**18–64**	4 (31)	0	0	1 (100)	0	0	0	0	2 (50.0)	7 (3.1)	
**> 64**	2 (15)	1 (16.7)	3 (75.0)	0	1 (100)	0	0	1 (33.3)	0	8 (33.3)	
**Gender**											
**Male**	7 (53.8)	3 (50.0)	3 (75.0)	1 (100)	1 (100)	5 (38.5)	1 (100)	3 (100)	1 (25.0)	25 (9.9)	8.4 (0.40)
**Female**	6 (46.2)	3 (50.0)	1 (25.0)	0	0	8 (61.5)	0	0	3 (75.0)	21 (7.9)	
**Residence**											
**Urban**	10 (77.0)	2 (33.0)	4 (100)	0	0	10 (77.0)	1 (100)	2 (67)	4 (100)	33 (8.4)	13.4 (0.10)
**Rural**	3 (23.0)	4 (67.0)	0	1 (100)	1 (100)	3 (23.0)	0	1 (33)	0	13 (10.2)	

*Untypable (not successfully genotyped).

### 3.4. Phylogenetic analysis of the norovirus genotypes

**Fig 1:**
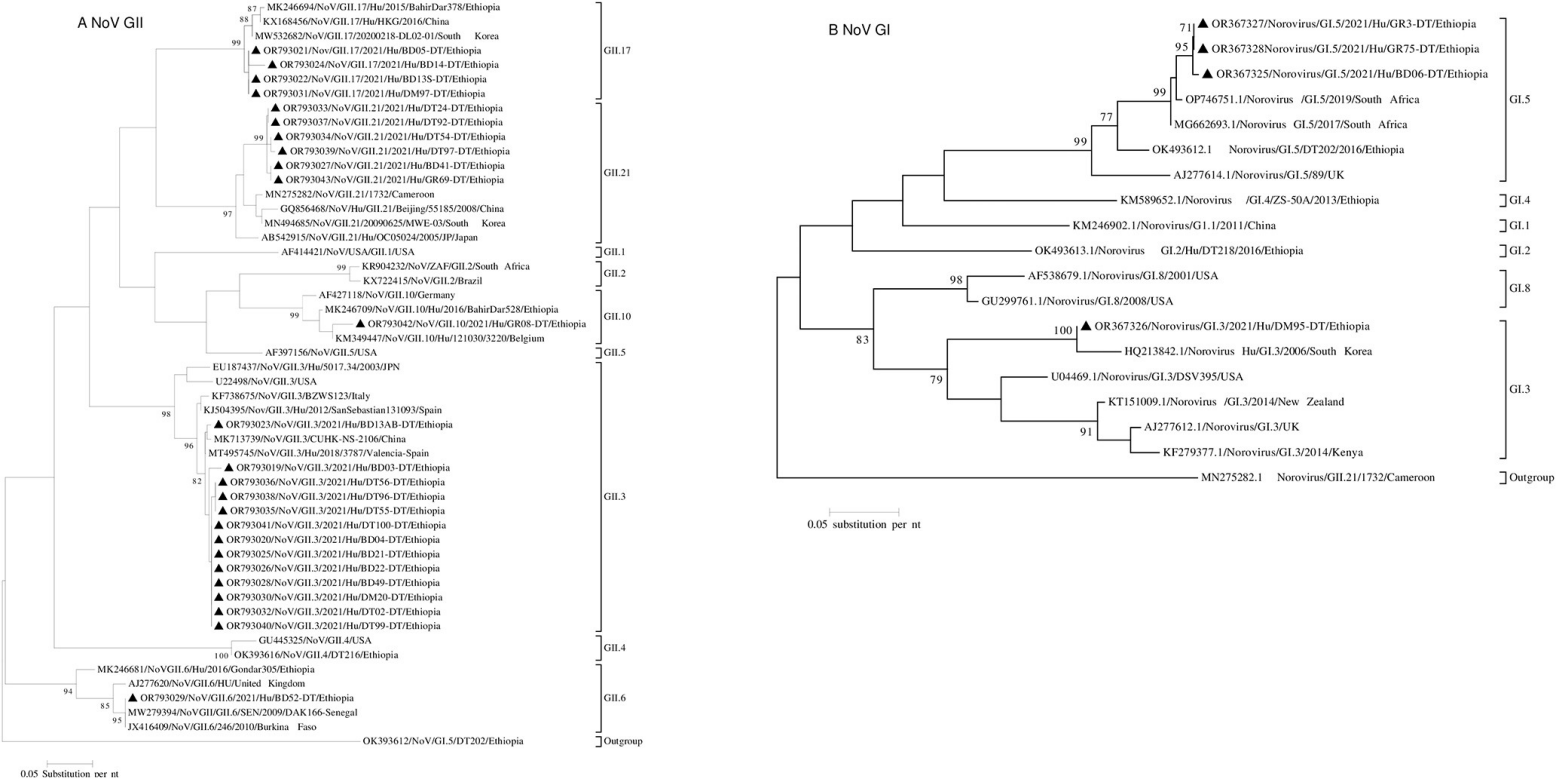
Phylogenetic trees of the norovirus GII (1A) and GI (1B). The trees were contracted based on the nucleotide sequences of the major capsid protein-coding region (VP1). Numbers at each node represent bootstrap values obtained with 1000 replicates (≥70 are shown). Isolates from this study are labeled with a black triangle.

The predominant genotype, GII.3, fell into two sub-clusters. In the first sub-cluster, an isolate from Bahir Dar (OR793023/NoV/GII.3/2021/Hu/BD13AB) showed close relatedness with a strain from China. In the second sub-cluster, the remaining twelve Gll.3 genotypes were grouped together and showed distinct clustering compared with reference strains from Spain, Japan, USA, and Italy. All the GII.21 genotypes were clustered together with global references from Cameroon, China, Japan, and South Korea. The GII.17 genotype clustered with an isolate from Ethiopia reported in 2015 and other global references from South Korea and China. The GII.6 genotype showed close relatedness to reference strains from Senegal, Burkina Faso as well as strains from Ethiopia and United Kingdom (UK). The GII.10 genotype clustered with the reference strains reported from German, Belgium and Ethiopia (Fig 1A). All the three GI.5 NoV genotypes sequenced were in one cluster and were closely related to each other as well as to the two reference strains reported from South Africa. These strains were also distantly related with the reference strains reported from Ethiopia and UK. Similarly, the GI.3 genotype was more closely related to the reference strain reported from South Korea. Besides, our GI.3 strain was distantly related with the reference strains from USA, UK, New Zealand, and Kenya (Fig 1B).

## 4. Discussion

In the present study, a considerable number of NoV infections were observed in diarrheal patients, whose ages ranged from three months to eighty-five years. The fecal samples were collected from four sampling sites in the Amhara National Regional State, each of which included three health facilities. Despite the geographic proximity of the four sampling sites, statistically significant differences were noted in the positivity rate, genetic diversity, and distribution of NoVs circulating in the region.

In the present study, the majority of NoV isolates (63%) were successfully sequenced and genotyped, whereas the remaining one-third of NoV-positive samples were not well amplified and sequenced. The failure in genotyping these positive samples might be due to degradation of the nucleic acid during storage and the low viral load, as evidenced by the higher Ct-value > 30 among the non-sequenced amplicons. In our study, the GII genogroup was more frequently detected accounting for 82.6% of the positive cases. This observation is consistent with reports of several studies worldwide [9,10,12,25,26]. In total, seven distinct NoV genotypes (GI.3, GI.5, GII.3, GII.6, GII.10, GII.17, and GII.21) were identified. This finding revealed the circulation of diversified NoV genotypes in the study areas.

Norovirus genotypes GII.3, GII.21, and GII.17 were relatively the most frequently detected genotypes, with GII.3 being the most prevalent of all. This finding is in agreement with previous study in Ethiopia [27], and studies in China [20,28,29]. Although most of the previous studies were conducted among under-5 children, the diversity of NoV genotypes, with the predominance of non-GII.4 NoV genotypes in our study, was similar to reports from other previous studies in Ethiopia [12,26,27]. In addition, the predominance of GII.3 was also reported in previous studies from Tunisia, Malaysia, and Bhutan [30–32]. Norovirus GII.3 was also reported as the second-predominant genotype after GII.4 in studies in China and lower- and middle-income countries [33,34]. However, NoV GII.4, which was the predominant genotype responsible for most of the diarrheal outbreaks worldwide [9,20], was not detected in this study. The possible explanation for the absence of the non-GII.4 norovirus genotype in the present study might be either the total absence of this genotype in the study areas and sites during the study period or it might be among those samples that failed to be amplified and sequenced either due to high Ct values or degradation of the RNA because of long-term storage conditions or repeated free-thaw cycles during amplification. It might also be by chance and need further investigation.

Similar to previous studies in Ethiopia [26] and China [14] a considerable number of GII.17 genotypes were detected in this study. Unlike the previous studies done in Ethiopia [12,26], the GII.21 genotypes were identified in the present study. In accordance with our study, NoV GII.21 has been reported in studies from Bhutan and Cameroon [31,35,36]. Hence, the detection of the GII.21 genotype in our study is a new addition to the growing evidence of the genetic diversity of NoVs in Ethiopia.

In the current study, two distinct GI genotypes, GI.3 and GI.5, were identified. In agreement with our findings, both GI.5 and GI.3 were commonly identified by other previous studies in Ethiopia [12,26,27] and a systematic review conducted in Africa [9].

Many of the NoV sequences deposited in the GenBank from the east African region are short or scattered within the major capsid coding regions. Because of this, the phylogenetic analysis of the present study was performed by collecting sequence data published mainly from Western and Asian counties, including some available sequences from South Africa, Cameroon, Senegal, Burkina Faso, and Kenya. In the cases where sequences are available from previous studies in Ethiopia, the genotypes identified from the present study showed closer phylogenetic relatedness [12,26,27].

Comprehensive data on the distribution of NoV genotypes across different study settings is limited in Ethiopia. Previous studies conducted in 2015 and 2016 among under-5 children in northwest Ethiopia by taking samples from two distinct areas (180 km apart) revealed a similar genotyping distribution between the two geographic areas [12,26,27]. However, in the present study, we tried to investigate the genetic diversity and distribution of NoVs by including two additional sampling sites and considering all age groups.

In contrast to the findings from the above studies, the genetic diversity and distribution of NoVs were entirely different across the four study sites. The highest genetic diversity was observed at Bahir Dar sampling sites. The possible explanation might be due to the highest population dynamics that increased person-to-person contact-mediated infection in the Bahir Dar area. That is, Bahir Dar is the capital city of the Amhara National Regional State, where the inflation of the population, including foreigners, is high as compared to other study areas. In addition to this, the prevalence and genetic diversity were higher among the elderly and under-5 children. The possible explanation, primarily, might be due to the increased prevalence of NoV among the elderly (33.5%) and under-5 children (12.5%) as compared to adults. This might be explained by factors such as underdeveloped or reduced immune function in these age groups [37] as well as increased environmental exposure, especially among under-5 children [38]. In addition to this, most of the samples were collected around September and October, when most children can have an increase in contact-mediated infections due to the beginning of school in these months in Ethiopia. Hence, these age groups can be exposed to and develop a disease from any genotype of the virus. In order to evaluate the relevance of this observation, future studies may need to include parallel studies at multiple sampling sites across the country.

This study provided informative data on the distribution and genetic diversity of NoVs by analyzing samples collected from multiple sites and targeting patients of all ages with diarrhea. However, genotyping of NoV was performed by sequencing a portion of the major capsid region, and hence the presence of recombinant genotypes was not assessed. Although GIV is a rare genogroup, it was not considered in this study, which might underestimate the outcome. Moreover, the presence of a considerable number of un-typable NoV-positive samples might have underestimated the genetic diversity of NoV in the present study.

## 5. Conclusions

The magnitude of NoV infection among diarrheal patients was considerably high. Both GI and GII NoV genotypes were detected. The highest positivity rate and genetic diversity were noted for GII with the identification of GII.21 genotypes for the first time in Ethiopia. The genetic diversity of NoVs was significantly different across the four sampling sites, with the highest diversity noted in Bahir Dar. Besides, genetic diversity was variable among the different age groups. Most of the detected genotypes showed close phylogenetic relatedness to each other and previous genotypes from Ethiopia and elsewhere. Future surveillance studies are necessary to gain comprehensive data on the burden and genetic diversity of NoVs in Ethiopia.

## Supporting information

S1 Table List of primers and probes. (DOCX)

S1 File GI NoV genotype sequences. (DOCX)

S2 File GII NoV genotype sequences. (DOCX)

S1 Appendix Informed consent and or assent form. (DOCX)

## Abbreviations

AHRI: Armauer Hansen Research Institute
APHI: Amhara Public Health Institute
EPHI: Ethiopian Public Health Institute
GI: Genogroup I
GII: Genogroup II
MEGA: Molecular Evolutionary Genetic Analysis
NoVs: Noroviruses
ORF: Open reading frame
PBS: Phosphate buffered saline
RdRp: RNA-dependent RNA polymerase
RNA: Ribonucleic acid
RT-PCR: Reverse transcription polymerase chain reaction
UK: United Kingdom
USA: United States of America
VP1: Viral capsid protein 1
