# Supplementary material for: Genetic diversity and distribution of noroviruses among all age groups of patients with diarrhea in Amhara National Regional State, Ethiopia
Source: PLoS One. 2024 May 21;19(5):e0303887. doi: 10.1371/journal.pone.0303887 (PMC11108165; doi:10.1371/journal.pone.0303887)
Supplement: S1 Table — (DOCX) [file pone.0303887.s001.docx]

**Genetic diversity and distribution of noroviruses among all age groups of patients with diarrhea in Amhara National Regional State, Ethiopia**

Dessie Tegegne^1, 2*^, Aschalew Gelaw^1^, Dawit Hailu Alemayehu^3^, Tamrayehu Seyoum^3^, Dereje Leta^4^, Getachew Ferede^1^, Andargachew Mulu^3*^, Baye Gelaw^1^

**S1 Table. List of primers and probes**

| **Primers or probes** | **Sequence (5’–3’)** | **Polarity** | **Genogroups** | **Target gene** | **Amplicon size (bp)** | **Location (5’→3’)** | **References** |
| --- | --- | --- | --- | --- | --- | --- | --- |
| **Norovirus real-time RT-PCR** | | | | | | | |
| NV192 (s) | GCYATGTTCCGCTGGATGC | F | NoV-GI | VP1 | 98 | 5282–5300^k^ | (1, 2) |
| NV193 (as) | CGTCCTTAGACGCCATCATCA | R | NoV-GI | VP1 | 98 | 5379–5359^k^ |  |
| *TM9-LNA | YAK-TG+GACA+GGA+GATC+GC-BBQ | F | NoV-GI | VP1 | 98 | 5345–5359^k^ |  |
| COG2F | CARGARBCNATGTTYAGRTGGATGAG | F | NoV-GII | VP1 | 98 | 4988-5013 |  |
| COG2R | TCGACGCCATCTTCATTCACA | R | NoV-GII | VP1 | 98 | 5085-5065 |  |
| *RING2-TP | FAM-TGGGAGGGCGATCGCAATCT-BBQ | F | NoV-GII | VP1 | 98 | 5048–5067^m^ |  |
| **Norovirus genotyping or sequencing** | | | | | | | |
| G1SKF | CTGCCCGAATTYGTAAATGA | F | NoV-GI | VP1 | 330 | 5342–5361 | (3) |
| G1SKR | CCAACCCARCCATTRTACA | R | NoV-GI | VP1 | 330 | 5671–5653 |  |
| G2SKF | CNTGGGAGGGCGATCGCAA | F | NoV-GII | VP1 | 344 | 5046–5064 |  |
| G2SKR | CCRCCNGCATRHCCRTTRTACAT | R | NoV-GII | VP1 | 344 | 5389–5367 |  |

**Key:** Nucleotide sequences are shown with standard codes, for degenerate primers; Y= C or T; R= G or A; M= C or A; K= G or T; S= C or G; D=G, A, or T; V=G, C, or A; H=C, T or A and B= C or G or T. R = A,G ; Y = C,T; M = A,C ; K = G,T; S =C,G; W = A,T; H = A,C,T ; B = C,G,T ; V = A,C,G ; D = A,G,T; N = A,C,G,T; *= a probe, ^k^M87661 (NoV GI, Norwalk), ^m^X86557 (NoV GII, Lordsdale), NoV-GI = norovirus genogroup I, NoV-GII = Norovirus genogroup II, VP1 = viral protein 1

1. Kageyama T, Kojima S, Shinohara M, Uchida K, Fukushi S, Hoshino FB, et al. Broadly Reactive and Highly Sensitive Assay for Norwalk-Like Viruses Based on Real-Time Quantitative Reverse Transcription-PCR. *Journal of Clinical Microbiology*. 2003:1548-57.

2. Gelaw A, Pietsch C, Mann P, Liebert U. Molecular detection and characterisation of sapoviruses and noroviruses in outpatient children with diarrhoea in Northwest Ethiopia. Epidemiology & Infection. 2019;147.

3. Kojima S, Kageyama T, Fukushi S, Hoshino FB, Shinohara M, Uchida K, et al. Genogroup-specific PCR primers for detection of Norwalk-like viruses. *Journal of Virological methods*. 2002;100(1-2):107-14.
