# Supplementary material for: Genetic diversity and distribution of noroviruses among all age groups of patients with diarrhea in Amhara National Regional State, Ethiopia
Source: PLoS One. 2024 May 21;19(5):e0303887. doi: 10.1371/journal.pone.0303887 (PMC11108165; doi:10.1371/journal.pone.0303887)
Supplement: S1 File — (DOCX) [file pone.0303887.s002.docx]

**Genetic diversity and distribution of noroviruses among all age groups of patients with diarrhea in Amhara National Regional State, Ethiopia.**

Dessie Tegegne^1, 2*^, Aschalew Gelaw^1^, Dawit Hailu Alemayehu^3^, Tamrayehu Seyoum^3^, Dereje Leta^4^, Getachew Ferede^1^, Andargachew Mulu^3*^, Baye Gelaw^1^

**S1 Supporting Information. GI NoV genotype sequences**

**>** **OR367325/Norovirus/GI.5/2021/Hu/BD06-DT/Ethiopia**

TTGGCGCGAATGGCGCCGGTCAGCTTGTGCCGGAGGTTAATAATGGCTATCCACTGCCACTTGATCCAGTGGCGGGGGCTTCCACCGCCCTTGCTACTGCTGGACAAGTTAATATGATTGACCCATGGATCTTTAATAATTTTGTCCAGGCCCCCCAGGGCGAATTCACTATTTCCCCAAACAACACTCCCGGCGATGTCCTGTTTGATTTACAATTAGGTCCACATCTAAATCCTTTTCTAGCACATTTGTCTCAGATGTATAATGGCTGGGTTGG

**>** **OR367326/Norovirus/GI.3/2021/Hu/DM95-DT/Ethiopia**

ATGGCACCAGTGGTGCCGGCCAGCTGGTACCAGAGGCAAACACAGCTGAGCCTATTGCTATGGATCCAGTAGTTGGCGCTGCCACAGCAGTCGCCACTGCTGGTCAAGTAAATATGATTGACCCCTGGATTATGATAATTTTGTTCAAGCACCTCAAGGAGAGTTTACAATTTCACCCAATAATACACCTGGTGATATTTTATTTGATTTGCAATTAGGACCTCAATTAAACCCCTTTTTGTCCCATTTAGCACAAATGTATAATGG

**> OR367327/Norovirus/GI.5/2021/Hu/GR3-DT/Ethiopia**

TTGGCGCGAATGGCGCCGGTCAGCTTGTGCCGGAGGTTAATAATGGCTATCCACTGCCACTTGATCCGTGGCGGGGGCTTCCACCGCCCTTGCTACTGCTGGACAAGTTAATATGATTGACCCATGGATCTTTAATAATTTTGTCCAGGCCCCCCAGGGCGAATTCACTATTTCCCCAAACAACACCCCCGGCGATGTCCTGTTTTTTACAATTAGGTCCACATCTAAATCCTTTTCTAGCACATTTGTCTCAGATGTATAATGGCTGGGTTGG

**>** **OR367328Norovirus/GI.5/2021/Hu/GR75-DT/Ethiopia**

TTGGCGCGAATGGCGCCGGTCAGCTTGTGCCGGAGGTTAATAATGGCTATCCACTGCCACTTGATCCAGTGGCGGGGGCTTCCACCGCCCTTGCTACTGCTGGACAAGTTAATATGATTGACCCATGGATCTTTAATAATTTTGTCCAGGCCCCCCAGGGCGAATTCACTATTTCCCCAAACAACACCCCCGGCGATGTCCTGTTTGATTTACAATTAGGTCCACATCTAAATCCTTTTCTAGCACATTTGTCTCAGATGTATAATGGCTGGGTTGG
