# Supplementary material for: Genetic diversity and distribution of noroviruses among all age groups of patients with diarrhea in Amhara National Regional State, Ethiopia
Source: PLoS One. 2024 May 21;19(5):e0303887. doi: 10.1371/journal.pone.0303887 (PMC11108165; doi:10.1371/journal.pone.0303887)
Supplement: S2 File — (DOCX) [file pone.0303887.s003.docx]

**Genetic diversity and distribution of noroviruses among all age groups of patients with diarrhea in Amhara National Regional State, Ethiopia.**

Dessie Tegegne^1, 2*^, Aschalew Gelaw^1^, Dawit Hailu Alemayehu^3^, Tamrayehu Seyoum^3^, Dereje Leta^4^, Getachew Ferede^1^, Andargachew Mulu^3*^, Baye Gelaw^1^

**S2 Supporting Information. GII NoV genotype with GenBank accession numbers and sequences**

**>OR793019/Norovirus GII /GII.3/2021/Hu/BD03-DT/Ethiopia**

CGAATGACGCTGCTCCATCTAATGATGGTGCCGCCGGCCTCGTGCCAGACATCAATAAGAAGGCAATGGCGCTAGATCCAGTGGCGGGTGCAGCGATAGCAGCACCCCTTACTGGCCAGCAAAATATAATAGATCCCTGGATTATGAACAATTTTGTGCAAGCACCTGGTGGTGAGTTTACAGTGTCACCTAGGAACTCCCCCGTGAAGTACTTCTAAATTTAGAATTAGGCCCAGAAATAAATCCCTACCTGGCGCACCTTGCTAGGATGTAATGGTTATGCC

**> OR793020/Norovirus GII /GII.3/2021/Hu/BD04-DT/Ethiopia**

CGAATGACGCTGCTCCATCTAATGATGGTGCCGCCGGCCTCGTGCCAGAGATCAATAATGAGGCAATGGCGCTAGATCCAGTGGCGGGTGCAGCGATAGCAGCACCCCTTACTGGCCAGCAAAATATAATAGATCCCTGGATTATGAACAATTTTGTGCAAGCACCTGGTGGTGAGTTTACAGTGTCACCTAGGAACTCCCCCGGTGAAGTACTTCTAAATTTAGAATTAGGCCCAGAAATAAATCCCTACCTGGCGCACCTTGCTAGGATGTACAATGGTTATGCC

**> OR793021/Norovirus/GII.17/2021/Hu/BD05-DT/Ethiopia**

CGAATGACGCCGCTCCATCTAACGATGGTGCTGCCGGTCTCGTACCAGAGGGCAACAACGAGACCCTTCCCCTAGAACCAGTTGCGGGCGCAGCCATAGCCGCACCCGTCACTGGCCAAAATAATATAATTGACCCCTGGATTAGAACAAATTTTGTGCAAGCACCAAATGGAGAGTTCACAGTGTCACCCAGAAACTCTCCTGGAGAAATTTTATTAAATTTAGAATTGGGCCCTGATTTGAACCCTTATCTGGCTCATTTGTCAAGGATGTACAATGGTTATGCC

**>OR793022/Norovirus/GII.17/2021/Hu/BD13S-DT/Ethiopia**

CGAATGACGCCGCTCCATCTAACGATGGTGCTGCCGGTCTCGTACCAGAGGGCAACAACGAGACCCTTCCCCTAGAACCAGTTGCGGGCGCAGCCATAGCCGCACCCGTCACTGGCCAAAATAATATAATTGACCCCTGGATTAGAACAAATTTTGTGCAAGCACCAAATGGAGAGTTCACAGTGTCACCCAGAAACTCTCCTGGAGAAATTTTATTAAATTTAGAATTGGGCCCTGATTTGAACCCTTATCTGGCTCATTTGTCAAGGATGTACAATGGTTATGCC

**>OR793023/Norovirus/GII.3/2021/Hu/BD13AB-DT/Ethiopia**

CGAATGACGCTGCTCCATCTAATGATGGTGCCGCCGGCCTCGTACCAGAGATCAATAATGAGGCAATGGCGCTAGATCCAGTGGCGGGTGCAGCGATAGCAGCACCCCTTACTGGCCAGCAAAATATAATAGATCCCTGGATTATGAACAATTTTGTGCAAGCACCTGGTGGTGAGTTTACAGTGTCACCTAGGAACTCACCCGGTGAAGTACTTCTAAATTTAGAATTAGGCCCAGAAATAAATCCCTACCTGGCGCACCTTGCTAGGATGTACAATGGTTATGCC

**>OR793024/Norovirus/GII.17/2021/Hu/BD14-DT/Ethiopia**

CGAATGACGCCGCTCCATCTAACGATGGTGCTGCCGGTCTCGTACCAGAGGGCAACAACGGGACCCTTCCCCTAGAACCAATAGCGGGCGCAGCCATAGCCGCACCCGTCACTGGCCAAAATAATATAATTGACCCCTGGATTAGAACAAATTTTGTGCAATCACCAAATGGAGAGTTCACAGTGTCACCCAGAAACTCTCCTGGAGAAATTTTATTAAATTTAGAATTGGGCCCTGATTTGAACCCTTATCTGGCTCATTTGTCAAGGATGTACAATGGTTATGCC

**>OR793025/Norovirus/GII.3/2021/Hu/BD21-DT/Ethiopia**

CGAATGACGCTGCTCCATCTAATGATGGTGCCGCCGGCCTCGTGCCAGAGATCAATAATGAGGCAATGGCGCTAGATCCAGTGGCGGGTGCAGCGATAGCAGCACCCCTTACTGGCCAGCAAAATATAATAGATCCCTGGATTATGAACAATTTTGTGCAAGCACCTGGTGGTGAGTTTACAGTGTCACCTAGGAACTCCCCCGGTGAAGTACTTCTAAATTTAGAATTAGGCCCAGAAATAAATCCCTACCTGGCGCACCTTGCTAGGATGTACAATGGTTATGCC

**>OR793026/Norovirus/GII.3/2021/Hu/BD22-DT/Ethiopia**

CGAATGACGCTGCTCCATCTAATGATGGTGCCGCCGGCCTCGTGCCAGAGATCAATAATGAGGCAATGGCGCTAGATCCAGTGGCGGGTGCAGCGATAGCAGCACCCCTTACTGGCCAGCAAAATATAATAGATCCCTGGATTATGAACAATTTTGTGCAAGCACCTGGTGGTGAGTTTACAGTGTCACCTAGGAACTCCCCCGGTGAAGTACTTCTAAATTTAGAATTAGGCCCAGAAATAAATCCCTACCTGGCGCACCTTGCTAGGATGTACAATGGTTATGCC

**>OR793027/Norovirus/GII.21/2021/Hu/BD41-DT/Ethiopia**

CGAATGACGCCGCCCCATCTAATGATGGTGCTACAGGTCTCGTACCAGAGAACAACACTGAGACCTTGCCCCTTGAACCCGTGGCCGGAGCGGCAATTGCTGCCCCTGTTACGGGCCAAAATAATATAATTGATCCCTGGATTAGAAGCAATTTTGTGCAGGCACCTAATGGTGAATTCACAGTTTCCCCTAGAAATTCTCCTGGTGAAATCTTAATGAATTTGGAGTTAGGGCCAGATCTTAATCCATATTTAGCCCACCTTTCAAGAATGTACAATGGTTATGCC

**>OR793028/Norovirus/GII.3/2021/Hu/BD49-DT/Ethiopia**

CGAATGACGCTGCTCCATCTAATGATGGTGCCGCCGGCCTCGTGCCAGAGATCAATAATGAGGCAATGGCGCTAGATCCAGTGGCGGGTGCAGCGATAGCAGCACCCCTTACTGGCCAGCAAAATATAATAGATCCCTGGATTATGAACAATTTTGTGCAAGCACCTGGTGGTGAGTTTACAGTGTCACCTAGGAACTCCCCCGGTGAAGTACTTCTAAATTTAGAATTAGGCCCAGAAATAAATCCCTACCTGGCGCACCTTGCTAGGATGTACAATGGTTATGCC

**>OR793029/Norovirus/GII.6/2021/Hu/BD52-DT/Ethiopia**

CGAATGACGCTGCTCCATCGAATGATGGTGCTGCCAACCTCGTACCAGAGGCCAACAATGAGGTTATGGCACTTGAACCGGTGGTGGGAGCCTCAATCGCAGCTCCTGTTGTCGGTCAGCAAAATATAATTGACCCCTGGATTAGAGAAAATTTTGTCCAAGCACCACAGGGCGAGTTCACTGTCTCACCAAGGAACTCGCCCGGTGAAATGCTTTTAAACCTTGAATTGGGCCCAGAACTCAACCCCTACTTGAGCCATTTATCCCGCATGTACAATGGTTATGCC

**>OR793030/Norovirus/GII.3/2021/Hu/DM20-DT/Ethiopia**

CGAATGACGCTGCTCCATCTAATGATGGTGCCGCCGGCCTCGTGCCAGAGATCAATAATGAGGCAATGGCGCTAGATCCAGTGGCGGGTGCAGCGATAGCAGCACCCCTTACTGGCCAGCAAAATATAATAGATCCCTGGATTATGAACAATTTTGTGCAAGCACCTGGTGGTGAGTTTACAGTGTCACCTAGGAACTCCCCCGGTGAAGTACTTCTAAATTTAGAATTAGGCCCAGAAATAAATCCCTACCTGGCGCACCTTGCTAGGATGTACAATGGTTATGCC

**>OR793031/Norovirus/GII.17/2021/Hu/DM97-DT/Ethiopia**

CGAATGACGCCGCTCCATCTAACGATGGTGCTGCCGGTCTCGTACCAGAGGGCAACAACGAGACCCTTCCCCTAGAACCAGTTGCGGGCGCAGCCATAGCCGCACCCGTCACTGGCCAAAATAATATAATTGACCCCTGGATTAGAACAAATTTTGTGCAAGCACCAAATGGAGAGTTCACAGTGTCACCCAGAAACTCTCCTGGAGAAATTTTATTAAATTTAGAATTGGGCCCTGATTTGAACCCTTATCTGGCTCATTTGTCAAGGATGTACAATGGTTATGCC

**>OR793032/Norovirus/GII.3/2021/Hu/DT02-DT/Ethiopia**

CGAATGACGCTGCTCCATCTAATGATGGTGCCGCCGGCCTCGTGCCAGAGATCAATAATGAGGCAATGGCGCTAGATCCAGTGGCGGGTGCAGCGATAGCAGCACCCCTTACTGGCCAGCAAAATATAATAGATCCCTGGATTATGAACAATTTTGTGCAAGCACCTGGTGGTGAGTTTACAGTGTCACCTAGGAACTCCCCCGTGAAGTACTTCTAAATTTAGAATTAGGCCCAGAAATAAATCCCTACCTGGCGCACCTTGCTAGGATGTGGTTATGCC

**> OR793033/Norovirus/GII.21/2021/Hu/DT24-DT/Ethiopia**

CGAATGACGCCGCCCCATCTAATGATGGTGCTACAGGTCTCGTACCAGAGAACAACACTGAGACCTTGCCCCTTGAACCCGTGGCCGGAGCGGCAATTGCTGCCCCTGTTACGGGCCAAAATAATATAATTGATCCCTGGATTAGAAGCAATTTTGTGCAGGCACCTAATGGTGAATTCACAGTTTCCCCTAGAAATTCTCCTGGTGAAATTTTAATGAATTTGGAGTTAGGGCCAGATCTTAATCCATATTTAGCCCACCTTTCAAGAATGTACAATGGTTATGCC

**> OR793034/Norovirus/GII.21/2021/Hu/DT54-DT/Ethiopia**

CGAATGACGCCGCCCCATCTAATGATGGTGCTACAGGTCTCGTACCAGAGAACAACACTGAGACCTTGCCCCTTGAACCCGTGGCCGGAGCGGCAATTGCTGCCCCTGTTACGGGCCAAAATAATATAATTGATCCCTGGATTAGAAGCAACTTTGTGCAGGCACCTAATGGTGAATTCACAGTTTCCCCTAGAAATTCTCCTGGTGAAATTTTAATGAATTTGGAGTTAGGGCCAGATCTTAATCCATATTTAGCCCACCTTTCAAGAATGTACAATGGTTATGCC

**> OR793035/Norovirus/GII.3/2021/Hu/DT55-DT/Ethiopia**

CGAATGACGCTGCTCCATCTAATGATGGTGCCGCCGGCCTCGTGCCAGAGATCAAAAATGAGGCAATGGCGCTAGATCCAGTGGCGGGTGCAGCGATAGCAGCACCCCTTACTGGCCAGCAAAATATAATAGATCCCTGGATTATGAACAATTTTGTGCAAGCACCTGGTGGTGAGTTTACAGTGTCACCTAGGAACTCCCCCGGTGAAGTACTTCTAAATTTAGAATTAGGCCCAGAAATAAATCCCTACCTGGCGCACCTTGCTAGGATGTACAATGGTTATGCC

**> OR793036/Norovirus/GII.3/2021/Hu/DT56-DT/Ethiopia**

CGAATGACGCTGCTCCATCTAATGATGGTGCCGCCGGCCTCGTGCCAGAGATCAAAAATGAGGCAATGGCGCTAGATCCAGTGGCGGGTGCAGCGATAGCAGCACCCCTTACTGGCCAGCAAAATATAATAGATCCCTGGATTATGAACAATTTTGTGCAAGCACCTGGTGGTGAGTTTACAGTGTCACCTAGGAACTCCCCCGGTGAAGTACTTCTAAATTTAGAATTAGGCCCAGAAATAAATCCCTACCTGGCGCACCTTGCTAGGATGTACAATGGTTATGCC

**> OR793037/Norovirus/GII.21/2021/Hu/DT92-DT/Ethiopia**

CGAATGACGCCGCCCCATCTAATGATGGTGCTACAGGTCTCGTACCAGAGAACAACACTGAGACCTTGCCCCTTGAACCCGTGGCCGGAGCGGCAATTGCTGCCCCTGTTACGGGCCAAAATAATATAATTGATCCCTGGATTAGAAGCAATTTTGTGCAGGCACCTAATGGTGAATTCACAGTTTCCCCTAGAAATTCTCCTGGTGAAATTTTAATGAATTTGGAGTTAGGGCCAGATCTTAATCCATATTTAGCCCACCTTTCAAGAATGTACAATGGTTATGCC

**> OR793038/Norovirus/GII.3/2021/Hu/DT96-DT/Ethiopia**

CGAATGACGCTGCTCCATCTAATGATGGTGCCGCCGGCCTCGTGCCAGAGATCAAAAATGAGGCAATGGCGCTAGATCCAGTGGCGGGTGCAGCGATAGCAGCACCCCTTACTGGCCAGCAAAATATAATAGATCCCTGGATTATGAACAATTTTGTGCAAGCACCTGGTGGTGAGTTTACAGTGTCACCTAGGAACTCCCCCGGTGAAGTACTTCTAAATTTAGAATTAGGCCCAGAAATAAATCCCTACCTGGCGCACCTTGCTAGGATGTACAATGGTTATGCC

**> OR793039/Norovirus/GII.21/2021/Hu/DT97-DT/Ethiopia**

CGAATGACGCCGCCCCATCTAATGATGGTGCTACAGGTCTCGTACCAGAGAACAACACTGAGACCTTGCCCCTTGAACCCGTGGCCGGAGCGGCAATTGCTGCCCCTGTTACGGGCCAAAATAATATAATTGATCCCTGGATTAGAAGCAACTTTGTGCAGGCACCTAATGGTGAATTCACAGTTTCCCCTAGAAATTCTCCTGGTGAAATCTTAATGAATTTGGAGTTAGGGCCAGATCTTAATCCATATTTAGCCCACCTTTCAAGAATGTACAATGGTTATGCC

**> OR793040/Norovirus/GII.3/2021/Hu/DT99-DT/Ethiopia**

CGAATGACGCTGCTCCATCTAATGATGGTGCCGCCGGCCTCGTGCCAGAGATCAATAATGAGGCAATGGCGCTAGATCCAGTGGCGGGTGCAGCGATAGCAGCACCCCTTACTGGCCAGCAAAATATAATAGATCCCTGGATTATGAACAATTTTGTGCAAGCACCTGGTGGTGAGTTTACAGTGTCACCTAGGAACTCCCCCGGTGAAGTACTTCTAAATTTAGAATTAGGCCCAGAAATAAATCCCTACCTGGCGCACCTTGCTAGGATGTACAATGGTTATGCC

**> OR793041/Norovirus/GII.3/2021/Hu/DT100-DT/Ethiopia**

CGAATGACGCTGCTCCATCTAATGATGGTGCCGCCGGCCTCGTGCCAGAGATCAATAATGAGGCAATGGCGCTAGATCCAGTGGCGGGTGCAGCGATAGCAGCACCCCTTACTGGCCAGCAAAATATAATAGATCCCTGGATTATGAACAATTTTGTGCAAGCACCTGGTGGTGAGTTTACAGTGTCACCTAGGAACTCCCCCGTGAAGTACTTCTAAATTTAGAATTAGGCCCAGAAATAAATCCCTACCTGGCGCACCTTGCTAGGATGTACAATGGTTATGCC

**> OR793042/Norovirus/GII.10/2021/Hu/GR08-DT/Ethiopia**

CGCCGCTCCATCTAGTGATGGTGCAGCCGGCCTCGTGCCAGAAAGTAATACTGAGGTTATGGCTCTTGAACCTGTAGCTGGGGCGTCTTTAGCTGCCCCTGTGACGGGTCAAACAAATATAATTGACCCATGGATAAGAATGAACTTTGTTCAAGCCCCAAATGGAGAGTTTACTGTTTCCCCAAGAAATTCCCCTGGAGAAGTACTCCTAAATTTGGAACTGGGTCCTGAATTAAATCCTTATCTGGCACATTTATCCAGGATGTACAATGGCTATGCT

**> OR793043/Norovirus/GII.21/2021/Hu/GR69-DT/Ethiopia**

CGAATGACGCCGCCCCATCTAATGATGGTGCTACAGGTCTCGTACCAGAGAACAACACTGAGACCTTGCCCCTTGAACCCGTGGCCGGAGCGGCAATTGCTGCCCCTGTTACGGGCCAAAATAATATAATTGATCCCTGGATTAGAAGCAATTTTGTGCAGGCACCTAATGGTGAATTCACAGTTTCCCCTAGAAATTCTCCTGGTGAAATCTTAATGAATTTGG
