# Supplementary material for: Genetic diversity and distribution of noroviruses among all age groups of patients with diarrhea in Amhara National Regional State, Ethiopia
Source: PLoS One. 2024 May 21;19(5):e0303887. doi: 10.1371/journal.pone.0303887 (PMC11108165; doi:10.1371/journal.pone.0303887)
Supplement: S1 Appendix — (DOCX) [file pone.0303887.s004.docx]

**Genetic diversity and distribution of noroviruses among all age groups of patients with diarrhea in Amhara National Regional State, Ethiopia**

Dessie Tegegne^1, 2*^, Aschalew Gelaw^1^, Dawit Hailu Alemayehu^3^, Tamrayehu Seyoum^3^, Dereje Leta^4^, Getachew Ferede^1^, Andargachew Mulu^3*^, Baye Gelaw^1^

**S1 Appendix. Informed consent and or assent form**

Participant: Age---------- Sex------------ Unique code: -------------------

I have been informed about the research study entitled “Genetic diversity and distribution of noroviruses among all age groups of patients with diarrhea in Amhara National Regional State, Ethiopia” which is aimed to assess the genetic diversity and distribution of noroviruses among patients with diarrhea in the Amhara National Regional State, Ethiopia, that is going to be conducted from May 01/2021 to November 30/2021.

I have been informed and/or seen that the project is approved by the University of Gondar Institutional Ethical Review Board and given a permission to conduct the study. As the study participant and/or a parent, the purpose of the study had been briefly explained to me. Moreover, I have been well informed of my right to refuse, decline to cooperate and or drop out of the study if I want and none of my actions will have any bearing at all on my overall health care services.

It is therefore with full understanding of the situation that I agreed to give the informed consent/assent form voluntarily to the researcher to give specimens (stool) for the study. I have the opportunity to ask questions about the project and received clarification to my satisfaction in a language I understand. I have been informed that participation in the study has no any specific harm and or benefits, but an indirect benefit as a community.

I have read this information (had the information read to me). I have been given enough time to think over before I signed this informed consent/assent. It is therefore, with full understanding of the situation that I gave my informed consent/assent and cooperate to participate fully in the course of the study.

Participant /Parent/Guardian: Name: __________signature ______________ Date: _______

Researcher/Data collector: Name: __________signature ______________ Date: ________
